# Site-specific π-clamp-mediated radiosynthesis of ^68^Ga and ^18^F PET radiopharmaceuticals†

**DOI:** 10.1039/d4cc05223d

**Published:** 2024-12-09

**Authors:** Thomas T. C. Yue, Jin Hui Teh, Eric Aboagye, Michelle T. Ma, Truc T. Pham, Nicholas J. Long

**Affiliations:** a Department of Chemistry, Molecular Sciences Research Hub, Imperial College London White City Campus, Wood Lane London W120BZ UK n.long@imperial.ac.uk +44 (0)20 7594 5804 +44 (0)20 7594 5781; b School of Biomedical Engineering and Imaging Sciences, King's College London 4th Floor Lambeth Wing, St. Thomas’ Hospital London SE17EH UK truc.pham@kcl.ac.uk; c Department of Surgery & Cancer, Imperial Centre for Translational and Experimental Medicine, Imperial College London Hammersmith Campus UK

## Abstract

The π-clamp-mediated conjugation method, which enables site-specific modification of cysteine residues, is a promising strategy for developing well-defined radiolabelled biomolecules for positron emission tomography (PET) imaging. We have applied this method to site-specifically attach the macrocyclic chelators “NODA” and “NODAGA” to the somatostatin receptor 2-targeted peptide, octreotate. The resulting novel NODA-octreotate and NODAGA-octreotate compounds can be radiolabelled with either [^18^F]AlF^−^ or [^68^Ga]Ga^3+^ respectively. *In vivo* PET imaging shows that the [^68^Ga]Ga^3+^-labelled derivative exhibits high stability and favourable pharmacokinetic properties.

## Abstract 2

The π-clamp-mediated conjugation method, which enables site-specific modification of cysteine residues, is a promising strategy for developing well-defined radiolabelled biomolecules for positron emission tomography (PET) imaging.

## Graphical abstract

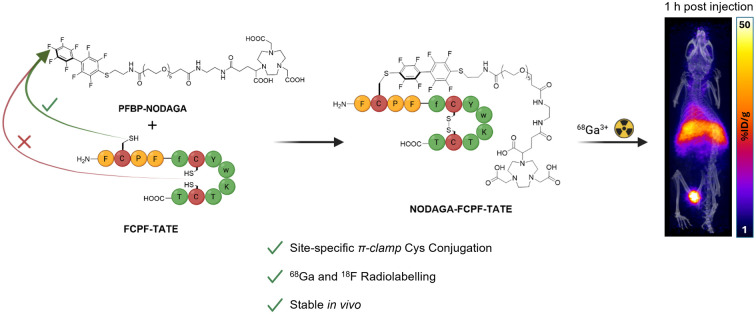

Site-selective protein modification allows the precise installation of different functional entities such as drugs and radioactive labels onto biomolecules.^1–3^ This strategy produces well-defined bioconjugates, and enables fine-tuning of biomolecular properties.^3,4^ One significant application of this approach is in positron emission tomography (PET) imaging, where site-selective modification and radiolabelling can lead to improved imaging quality and accuracy, facilitating better diagnosis and monitoring of diseases.^5,6^

Cysteine (Cys) residues are amongst the most useful motifs for protein modification due to the high nucleophilicity of the thiol group. However, traditional Cys-based conjugation strategies utilising electrophilic reagents such as maleimides lack regioselectivity when multiple reactive Cys residues are available, resulting in ill-defined, heterogenous mixtures.^7^ Perfluoroaryl reagents are well known to undergo aromatic substitution reactions with Cys residues and the resulting conjugates are highly stable.^8^ Zhang *et al.* have demonstrated that regioselective Cys modification can be achieved using perfluoroaryl reagents through a “π-clamp” approach.^9^ This method utilises a four amino acid motif, Phe-Cys-Pro-Phe (FCPF), where the Cys thiol undergoes rapid and site-selective conjugation with perfluoroaryl reagents such as perfluorobiphenyl (PFBP) compounds in aqueous media. Notably, PFBP compounds do not react with other Cys residues, showcasing a high degree of regioselectivity and obviating the need for protecting group strategies or partial re-oxidation steps. This remarkable selectivity is facilitated by the hydrophobic interactions between the Phe side chains and the perfluoroaryl reagents, which creates a microenvironment that reduces the Cys thiol's p*K*_a_ and decreases the enthalpy of activation for the S_N_Ar reaction.^10^ Utilising the “π-clamp” strategy, perfluoroaryl-based reagents have been developed for the preparation of antibody–drug conjugates,^9^ protein–protein dimers,^11^ antibody-fluorophore conjugates,^12,13^ Re(i)- and Ir(iii)-based peptide conjugates for photodynamic therapy.^14,15^ This method is yet to be deployed in radiopharmaceuticals for receptor-targeted molecular PET imaging, where it could have tremendous utility.

Herein, we report a new class of 1,4,7-triazacyclononane-1,4-diacetate (NODA) and 1,4,7-triazacyclononane, 1-glutaric acid-4,7-acetic acid (NODAGA) based bifunctional chelates, PFBP-NODA (1) and PFBP-NODAGA (2) (Scheme 1), suitable for aluminium-[^18^F]fluoride ([^18^F]AlF) and [^68^Ga]Ga^3+^ radiolabelling respectively, while simultaneously facilitating site-specific conjugation *via* the “π-clamp” approach. Receptor-targeted peptide-based radiotracers have exhibited extraordinary clinical utility in cancer treatment.^16^ This includes octreotate (TATE) derivatives, [^68^Ga]Ga-DOTA-TATE and [^177^Lu]Lu-DOTA-TATE, which target the somatostatin receptor subtype 2 (SSTR2) and are now routinely clinically used to diagnose and treat patients with neuroendocrine cancers.^17–19^ Here, a TATE peptide modified with an N-terminal FCPF-tag was identified as a suitable target to demonstrate the utility of the bifunctional chelates. Importantly, TATE contains a pair of competing Cys residues which would serve as a proof of concept for site-specificity.

**Scheme 1:**
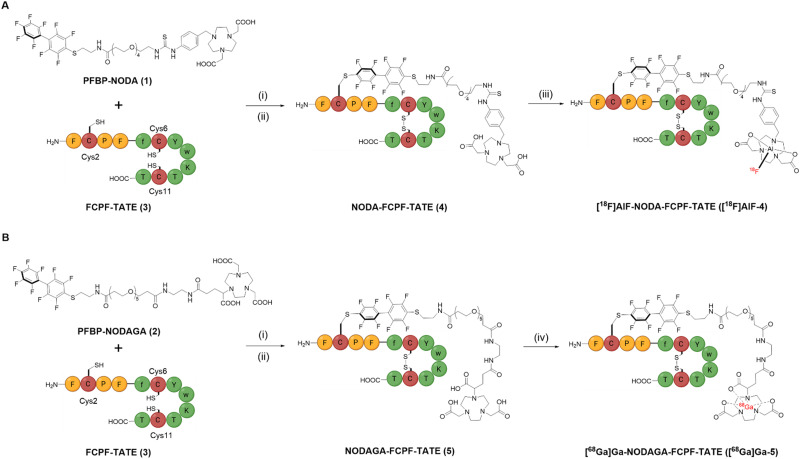
π-clamp mediated site-specific conjugation of FCPF-TATE (3) at Cys2 with (A) PFBP-NODA (1) and (B) PFBP-NODAGA (2), site-specificity confirmed by peptide mapping MS/MS experiment (Fig. S13, ESI†). Reaction conditions: (i) 20 mM TCEP, 0.2 M phosphate buffer, pH 8, 37 °C; (ii) 2,2′-dipyridyldisulphide, 2 M NH_4_OAc, pH 6, RT, 20 min; (iii) [^18^F]F^−^, AlCl_3_, 0.5 M NaOAc, pH 4.2, 100 °C, 20 min; (iv) [^68^Ga]Ga^3+^, 0.2 M NH_4_OAc, pH 6, 37 °C, 15 min.

The di-acetate and tri-acetate N-substituted 1,4,7-triazacyclononane derivatives, NODA and NODAGA, were attached to a PFBP unit, *via* polyethylene glycol (PEG) linkers (Scheme 1). We selected PEG linkers so as to (i) enhance the solubility of the PFBP derivatives in aqueous environments, and (ii) potentially prolong circulation of any resultant radiotracer in blood circulation. Synthetic procedures and characterisation data for the new bifunctional chelators, 1 and 2, are included in the ESI.†

We then interrogated the reaction of 1 and 2 with the functionalised peptide, FCPF-TATE (3) (Scheme 1). Reaction of 3 (1 mM) with 5 mol eq. of 1 or 2 (0.2 M phosphate buffer, 20 mM TCEP, 37 °C) resulted in formation of the desired reduced conjugates after just 30 min (Fig. S9, ESI†). Importantly, peptide mapping MS/MS experiments (Fig. S13, ESI†) revealed preferential modification at the FCPF Cys2 residue.

Surprisingly, formation of a minor second species, which has a *m/z* signal corresponding to a dual-conjugated product, was also observed after 1 h, and in increasing amounts over time (Fig. S9, ESI†). Peptide mapping revealed this to be a Cys6-modified species (Fig. S14, ESI†). Cys6 is immediately flanked by aromatic d-Phe5 and a Tyr7 residues, alongside Phe4 and d-Trp8 adjacent to these. We postulate that π-stacking between the FfCYw sequence and PFBP motifs leads to secondary regioselectivity in conjugation. Whilst undesirable for our purposes, we envisage that this “secondary” selectivity could be deployed in future, for applications in which two different payloads are site-selectively attached to peptides.

We next optimised the reaction conditions, including reducing the equivalence of the bifunctional chelator used and lowering the reaction temperature, to minimise the formation of dual conjugated products (Fig. S10, ESI†). In this fashion, higher yields of the desired product were achieved, confirming that 1 and 2 each preferentially react with Cys2. The reduced TATE conjugates were subsequently oxidised using 2,2′-dipyridyldisulphide and the resulting conjugates 4 and 5 were isolated *via* preparative-HPLC and characterised by high-resolution MS (Fig. S11 and S12, ESI†).

Conjugate 5 was radiolabelled with generator-produced [^68^Ga]Ga^3+^, by reaction of [^68^Ga]Ga^3+^ with 5 in aqueous solution at 37 °C for 15 min, at pH 6. This resulted in 79.5 ± 6.8% radiochemical conversion (RCC) (*n* = 4), as determined by radio-HPLC. [^68^Ga]Ga-NODAGA-FCPF-TATE ([^68^Ga]Ga-5) was simply purified using a Sep-Pak® C18 cartridge, yielding the conjugate in >99% radiochemical purity (RCP) and 49.7 ± 8.5% decay corrected (d.c.) radiochemical yield (RCY) (*n* = 3) (Fig. S15c, ESI†). Additionally, [^18^F]AlF radiolabelling of 4 was achieved, by incubating 4 with AlCl_3_ (2 mM) and [^18^F]Fluoride in aqueous solution at 100 °C for 20 min, at pH 4.2. This resulted in 74% RCC as determined by radio-HPLC analysis. [^18^F]AlF-NODA-FCPF-TATE ([^18^F]AlF-4) was purified using a Sep-Pak® C18 cartridge, and was recovered in 60% d.c. RCY and 93% RCP (Fig. S16b, ESI†).

We then elected to take forward the novel [^68^Ga]Ga-5 radiotracer for biological evaluation. [^68^Ga]Ga-5 was stable in human serum, with only 2% dissociation of the radiotracer observed after incubation at 37 °C for 5 h (Fig. S15d, ESI†). The dissociated activity had a radio-HPLC retention time coinciding with unchelated [^68^Ga]Ga^3+^, indicating that any instability is likely a result of ^68^Ga decomplexation rather than instability at the conjugation site. [^68^Ga]Ga-5 also demonstrated excellent stability in human serum solution containing 5 mM glutathione (GSH) for up to 4 h, with no observed thiol exchange or ^68^Ga-decomplexation (Fig. S15e, ESI†).

To evaluate the binding of [^68^Ga]Ga-5 towards its target SSTR2 receptor, the radiotracer was incubated with SSTR2-expressing AR42J rat pancreatic cells. After 60 min incubation at 37 °C, 25.3 ± 3.0% of added [^68^Ga]Ga-5 radioactivity was associated with AR42J cells (Fig. 1). The tracer was found to internalise rapidly, with only 20% of cell-associated radioactivity remaining surface-bound after 15 min. These observations are consistent with reports of other radiolabelled TATE derivatives.^20,21^ When AR42J cells were co-incubated with [^68^Ga]Ga-5 and an excess of unmodified octreotide peptide (200-fold), to “block” SSTR2 receptors and assess specificity of radiotracer uptake, significantly reduced amounts of radioactivity were associated with cells: uptake measured ≤ 6% uptake at all time points (*P* < 0.001 at 15 min, *P* < 0.01 at 60 min). This indicates that [^68^Ga]Ga-5 uptake in AR42J cells is largely mediated by SSTR2 receptor binding.

**Fig. 1:**
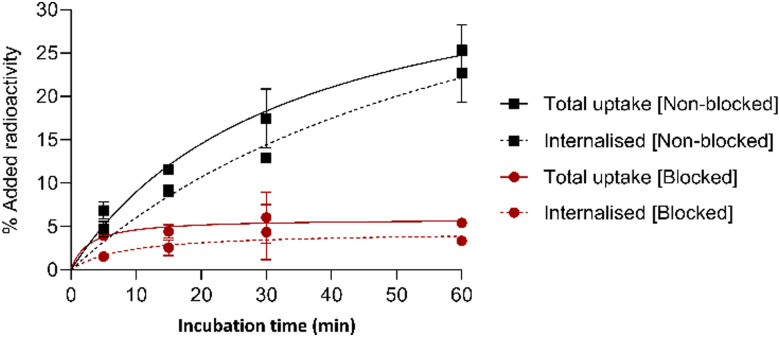
Uptake and internalisation of [^68^Ga]Ga-5 in SSTR2-positive AR42J cells (black), AR42J cells in the presence of 200-fold excess of octreotide (red) over time. Biological repeats were carried out in experimental triplicates (Mean ± SD, *n* = 3). See also Table S1 (ESI†).

Next, the biodistribution of [^68^Ga]Ga-5 in healthy mice was studied using PET/CT imaging alongside *ex vivo* tissue counting experiments. [^68^Ga]Ga-5 (0.8–1.5 MBq) was administered intravenously (*via* tail vein) to healthy BALB/c mice (*n* = 4) and dynamic PET/CT images were obtained over 1 h (Fig. 2A). Quantitative PET imaging analysis indicated that [^68^Ga]Ga-5 was cleared from the blood pool predominantly hepatically, with uptake in the liver and kidney measuring 24.61 ± 4.79% ID per g and 8.81 ± 0.73% ID per g respectively. This is in contrast to reported radiolabelled TATE derivatives, where renal clearance was predominantly observed.^20–23^ This is likely due to the comparatively high lipophilic nature of the radiotracer: the logD_7.4_ of [^68^Ga]Ga-5 measured −0.40 ± 0.03. In addition, relatively high concentrations of ^68^Ga radioactivity remained in circulation at 1 h p.i. (13.81 ± 1.61% ID per g), again, contrasting the behaviour of previously reported radiolabelled TATE bioconjugates.^20–23^ The radiotracer's high lipophilicity alongside its PEG5 linker likely play a role in extending its *in vivo* plasma half-life.^24^ The π-clamp strategy, not only offers a facile method for site-specific radiolabelling, but may also serve as an effective strategy for extending the pharmacokinetic half-life of radiotracers when desired.^25^ Following PET/CT imaging, the mice were culled, and organs weighed and counted for radioactivity (Fig. 2B). *Ex vivo* biodistribution analysis at 1 h p.i. corroborated with the PET/CT data: ^68^Ga radioactivity concentrations in the liver and kidneys measured 54.59 ± 22.47% ID per g and 9.58 ± 5.81% ID per g respectively, while the concentration of ^68^Ga radioactivity in the blood measured 27.24 ± 3.52% ID per g. Crucially, minimal skeletal uptake was observed, with uptake in the bone measuring 0.95 ± 0.46% ID per g, indicating the high stability of the ^68^Ga-NODAGA complex *in vivo.*

**Fig. 2:**
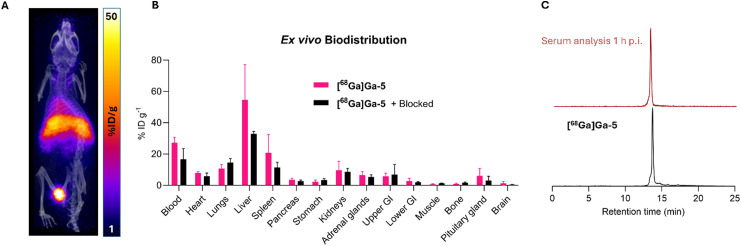
(A) Representative PET/CT maximum intensity projection (MIP) of healthy mice administered with [^68^Ga]Ga-5 at 1 h p.i. of radiotracer. (B) *Ex vivo* biodistribution of [^68^Ga]Ga-5 and [^68^Ga]Ga-5 co-administered with a blocking dose of octreotide (200-fold) in healthy mice at 1 h p.i. (Mean ± SD, *n* = 4). (C) RP-HPLC analysis of an *ex vivo* serum sample shows a single radioactive signal with retention time corresponding to [^68^Ga]Ga-5. See also Table S2 (ESI†).

Healthy organs such as the adrenal glands, pancreas and the pituitary glands are known to express SSTR2, and previous studies have shown that radiolabelled TATE derivatives could visualise these organs in mice.^21–23^ In order to assess the specificity of the radiotracer in these SSTR2-expressing organs, a second group of mice (*n* = 4) were co-administered with [^68^Ga]Ga-5 and a blocking dose of octreotide peptide (200-fold) to saturate endogenous SSTR2 receptors (Fig. 2B). In mice administered only [^68^Ga]Ga-5, there was considerable uptake of the tracer in SSTR2-expressing healthy organs such as the pancreas (3.49 ± 1.06% ID per g), adrenal glands (6.45 ± 2.28% ID per g) and pituitary glands (6.04 ± 4.82% ID per g), and these values all decreased for animals co-administered a blocking dose (mean difference: pancreas 0.85 ± 0.65% ID per g; adrenal glands 1.15 ± 1.36% ID per g; pituitary glands 2.94 ± 2.76% ID per g). However, these differences were not statistically significant in this experiment. This could be due to the high amounts of activity remaining in circulation at 1 h p.i., preventing accurate determination of specific organ uptake. Future studies could evaluate the biodistribution at later time points where lower amounts of radioactivity remain in circulation, offering a clearer picture of specific SSTR2-mediated tissue retention. *S*erum fractions from these mice were also collected at 1 h p.i. of the radiotracer, and analysed by RP-HPLC (Fig. 2C), with the resulting radiochromatogram revealing a single signal, coinciding with that of [^68^Ga]Ga-5. This indicates the high metabolic stability of the radiotracer *in vivo*, with no evidence of ^68^Ga-decomplexation or thiol exchange observed.

In summary, we present the first application of the π-clamp method for developing well-defined radiopharmaceuticals. The novel bifunctional chelates PFBP-NODA and PFBP-NODAGA were shown to selectively conjugate to the FCPF tag amidst other cysteines without requiring protecting group strategies, using a modified octreotate derivative. *In vitro* studies revealed that [^68^Ga]Ga-NODAGA-FCPF-TATE exhibited high affinity toward SSTR2 receptors. *In vivo*, [^68^Ga]Ga-NODAGA-FCPF-TATE demonstrated excellent stability, primarily exhibiting hepatic clearance and an extended circulation half-life compared to other radiolabelled TATE derivatives. These promising results open up new possibilities for using the facile π-clamp method with other biomolecules and radionuclides.

This research was supported by a Cancer Research UK Career Establishment Award (C63178/A24959) (to M. T. M.), the EPSRC Programme for Next Generation Molecular Imaging and Therapy with Radionuclides (EP/S032789/1, ‘MITHRAS’), the Wellcome Multiuser Equipment Radioanalytical Facility (212885/Z/18/Z), and a Schrodinger Studentship from Imperial College London (to T. T. C. Y.).

## Data availability

We can confirm that all the relevant research data is contained with the manuscript and electronic ESI.† No databases have been used and no references to such databases are contained in the manuscript or ESI.†

## Conflicts of interest

There are no conflicts to declare.

## Supplementary Material

CC-061-D4CC05223D-s001
