# Supplementary material for: Site-specific π-clamp-mediated radiosynthesis of 68Ga and 18F PET radiopharmaceuticals
Source: Chem Commun (Camb). 2024 Dec 9;61(4):732–5. doi: 10.1039/d4cc05223d (PMC11633826; doi:10.1039/d4cc05223d)
Supplement: CC-061-D4CC05223D-s001 [file CC-061-D4CC05223D-s001.pdf]

Supplementary Information (SI) for ChemComm.
This journal is © The Royal Society of Chemistry 2024

# Site-specific $\pi$-clamp-mediated radiosynthesis of $^{68}$Ga and $^{18}$F PET radiopharmaceuticals

Thomas T.C. Yue,[a,b] Jin Hui Teh,[a,c] Eric Aboagye,[c] Michelle T. Ma,[b] Truc T. Pham,*[b] Nicholas J. Long*[a]

[a] *Department of Chemistry, Molecular Sciences Research Hub, Imperial College London, White City Campus, Wood Lane, London W120BZ, UK. E-mail: n.long@imperial.ac.uk*

[b] *School of Biomedical Engineering and Imaging Sciences, King's College London, 4th Floor Lambeth Wing, St. Thomas' Hospital, London SE17EH, UK. E-mail: truc.pham@kcl.ac.uk*

[c] *Department of Surgery & Cancer, Imperial Centre for Translational and Experimental Medicine, Imperial College London, Hammersmith Campus, UK*

## Supporting Information

# Contents

Materials and Instrumentation ........ 2

Synthesis and characterisation data ........ 3

Peptide Conjugation ........ 6

Radiochemistry ........ 7

*In vitro* Analysis ........ 8

*In vivo* Analysis ........ 8

Supplementary Figures ........ 10

1.1 PFBP-NODA (1) and PFBP-NODAGA (2) Characterisation Data ........ 10

1.2 Peptide Conjugation ........ 14

1.3 Radiochemistry ........ 17

1.4 *In vitro* cell uptake ........ 18

1.5 *Ex vivo* biodistribution ........ 18

1.6 Dynamic PET/CT Imaging time-activity curves ........ 19

# Materials and Instrumentation

Chemicals were purchased from Merck Chemicals Ltd, Fluorochem Ltd or Tokyo Chemical Industry unless otherwise specified and used without further purification. Solvents for reactions were of reagent grade and were purchased from Merck Chemicals Ltd or VWR International and used without further purification. 18.2 MΩ water was used to prepare all buffers and aqueous solutions. Flash column chromatography was performed on a Biotage Isolera Prime advanced automated flash purification unit using SNAP Ultra C18 cartridges, unless otherwise stated. FCPF-Octretotate was purchased from PeptideSynthetics, Peptide Protein Research Ltd, UK. NCS-MP-NODA and $NH_2$-NODA-GA were purchased from CheMatech (Dijon, France).

$^{1}H$, $^{13}C\{^{1}H\}$, $^{19}F$ NMR spectra were recorded on a Bruker AV-400 at 298 K unless otherwise stated. Chemical shifts are reported in ppm, using the residual proton impurities in the solvents for $^{1}H$ and $^{13}C\{^{1}H\}$ measurements. $^{19}F$ chemical shifts were referenced (δ=0) to external $CFCl_3$ standards. Spectra were processed and analysed using MESTRELAB Mestrenova software. Peak multiplicities are abbreviated as follow: s = singlet, d = doublet, t = triplet, q = quartet, br = broad, m = multiplet.

High resolution mass spectrometry (HRMS) analyses were performed by Dr. Lisa Haigh and Dr. Malgorzata Puchnareqicz (Imperial College Department of Chemistry Mass Spectrometry service) using a using a Waters LCT Premier (ES-TOF) spectrometer. HPLC-MS analyses were conducted on a Waters 515 HPLC pump, 2998 photodiode array detector and 3100 mass detector, using either a Waters XSelect CSH C18 Column, 130 Å, 5 µm, 4.6 mm x 100 mm column or a Waters XBridge BEH C18 Column, 130 Å, 5 µm, 4.6 mm x 100 mm column. Liquid chromatography-tandem mass spectrometry (HPLC-MS/MS) analyses were performed on an Agilent 6545XT AdvenceBio LC/Q-Tof system using an Agilent Pursuit XRS C18 column, 5 µm, 150 x 4.6 mm, gradient: A = $H_2O$ + 0.1 % formic acid, B = MeCN + 0.1% formic acid, 0-1 min 20% B, 1-7 min 20-100% B, 7-8 min 100-20%, flow rate: 0.5 mL/min. MS conditions: positive ESI mass range 500-3000 m/z, temperature of drying gas = 350 ˚C, flow rate of drying gas = 10 L/min, pressure of nebuliser gas = 20 psi, capillary, fragmentor and octupole rf voltages were set at 5000, 350 and 750, respectively. Collision energy was set at 70 V. The Agilent MassHunter Bioconfirm software was used for the identification of FCPF-TATE modification. Preparative-HPLC was performed on an Agilent 1260 Infinity II Preparative LC System using an Agilent Pursuit XRs C18 column, 5 µm, 10 mm x 250 mm column.

Reverse-phase radioHPLC traces were acquired using an Agilent 1260 series HPLC system with an in-line radioactivity detector (LabLogic Systems Limited 1”NaI/PMT Detector). An Agilent ZORBAX Eclipse XDB-C18 column or a Gemini 5 µm C18 110 Å column was used with a mobile phase of 0.1% TFA H2O:MeCN 95:5 *v:v* to 5:95 *v:v* gradient at a flow rate of 1 mL/min. Instant thin layer chromatography (iTLC-SG) were obtained from Varian Medical Systems UK, Ltd. iTLC strips were visualised using a Lab Logic Dual Scan-RAM radio-TLC/HPLC Scanner.

# Synthesis and characterisation data

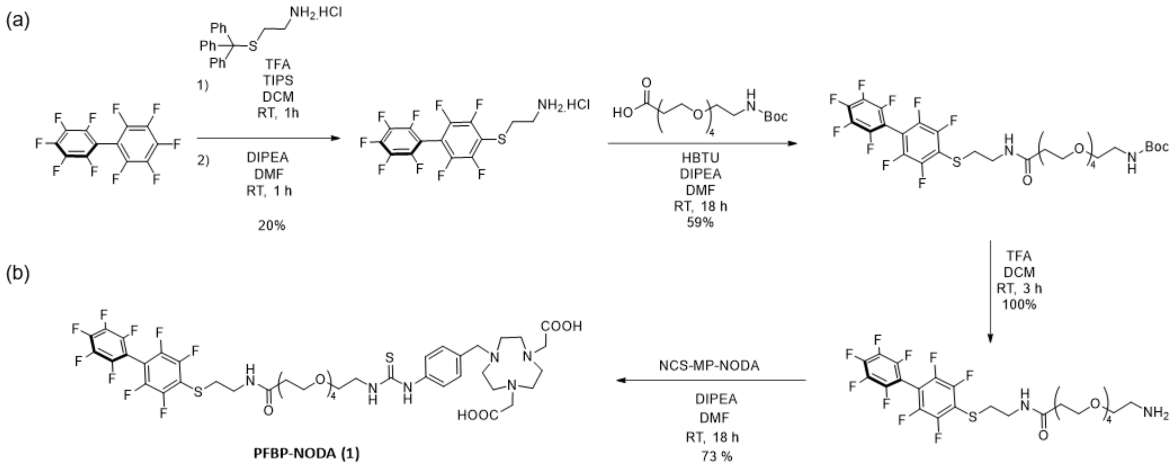


Scheme 1 Synthetic route to **PFBP-NODA (1)**

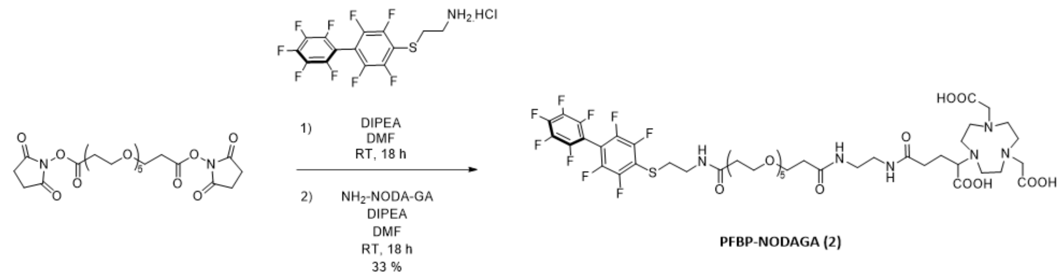


Scheme 2 Synthetic route to **PFBP-NODAGA (2)**

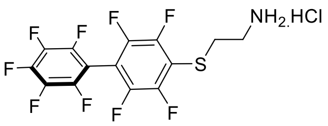


***PFBP-NH$_2$***: Trifluoroacetic acid (2 mL) was added dropwise to a solution of 2-Tritylthio-1-ethylamine hydrochloride (0.5 g, 1.41 mmol) and trisiopropyl silane (350 µL, 1.69 mmol) in $CH_2Cl_2$ (5 mL) and stirred at r.t. for 1 hr after which NMR analysis revealed complete deprotection of the trityl group. Volatiles were removed by passing a $N_2$ flow over the crude reaction before further drying *in vacuo.* The resulting residue was then dissolved in DMF (5 mL). Decafluorobiphenyl (3.75 g, 11.2 mmol) and N,N-diisopropylethylamine (1.5 mL, 11.2 mmol) was added and the resulting solution was stirred at r.t. for 1 h. The reaction was then quenched with 1M HCl and volatiles were removed in vacuo. The resulting residue was triturated in a mixture of MeCN and $H_2O$ (70:30) and filtered. The supernatant was dried in vacuo and purified *via* reversed-phase flash chromatography (C18, Biotage Sfar C18 Duo 100 Å 30 µm 12 g cartridge, A: 0.1 M $NH_4OAc$ B: 10 % 0.1 M $NH_4OAc$ in MeCN; 80 % A: 20% B → 100 % B) and lyophilised to yield the desired product as a white powder (120 mg, 0.28 mmol, 20 %).

$^1$H NMR (400 MHz, 298 K, $CDCl_3$) **δ**: 4.11 (br s, 2H, N*H$_2$*), 3.15 (t, 2H, RSC*H$_2$*CH$_2$NH$_2$), 2.95 (t, 2H, RSCH$_2$C*H$_2$*NH$_2$). $^{13}$C{$^1$H} NMR (101 MHz, 298 K, MeOD) **δ**: 148.5, 146.1, 145.5, 143.2, 142.9, 139.2, 136.7, 39.2, 33.8. $^{19}$F NMR (377 MHz, Chloroform-*d*) δ -132.42 - -132.56(m, 2F), -137.05 - -137.22 (m, 2F), -137.39 - -137.58 (m, 2F), -149.73 - - 149.93 (m, 1F), -160.21 - -160.42 (m, 2F). HRMS m/z (EOS-TOF, +ve): calcd. for $[M+H]^+$ $C_{14}H_7F_9NS^+$ 392.0150 found 392.0140

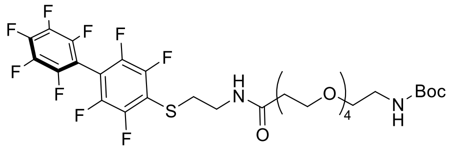


***PFBP-PEG$_4$-NHBoc***: To a solution of BocNH-PEG$_4$-COOH (92 mg, 0.24 mmol) in anhydrous DMF (5 mL) was added hexafluorophosphate benzotriazole tetramethyl uronium (HBTU) (101 mg, 0.27 mmol) and *N,N*-Diisopropylethylamine (106 µL, 0.60 mmol). The resulting solution was stirred at r.t. for 10 min, after which a solution containing PFBP-NH$_2$ (**4.1**) (124 mg, 0.27 mmol) and *N,N*-Diisopropylethylamine (190 µL, 1.08 mmol) in anhydrous DMF (3 mL) was added. The resulting reaction was stirred for 18 h at r.t. Volatiles were removed in vacuo and the residue purified by reverse phase column chromatography (C18, Biotage Sfar C18 Duo 100 Å 30 µm 12 g cartridge, A: 0.1 % TFA in $H_2O$ B: 0.1 % TFA in MeCN; 80 % A: 20 % B → 100 % B) and lyophilised to yield the desired product as a white powder (111 mg, 0.14 mmol, 59 %).

$^1$H NMR (400 MHz, 298 K, CD3CN) **δ**: 3.65 (t, *J* = 5.9 Hz, 2H), 3.61-3.52 (m, 12H), 3.45 (t, *J* = 5.6 Hz, 2H), 3.40 (q, *J* = 6.4 Hz, 2H), 3.22-3.12 (m, 4H), 2.40 (t, *J* = 6.0 Hz, 2H), 1.39 (s, 9H); $^{19}$F NMR (377 MHz, 298 K, CD3CN) **δ**: -132.66 to -136.34 (m, 2F), -139.37 to -139.59 (m, 2F), -139.81 to -140.04 (m, 2F), -152.60 (t, *J* = 20.2 Hz, 1F), -160.50 to -165.45 (m, 2F); HRMS m/z (EOS-TOF, +ve): calcd. for $[M+H]^+$ C30H35F9N2O7SNa 761.1919, found 761.1907.

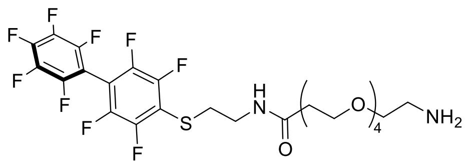


***PFBP-PEG$_4$-NH$_2$***: To a solution of PFBP-PEG$_4$-NHBoc (111 mg, 0.15 mmol) in $CH_2Cl_2$ (1 mL) was added trifluoroacetic acid (0.5 mL) and the resulting solution was stirred at r.t. for 3 h. Volatiles were removed by passing a $N_2$ flow over the crude reaction before further drying *in vacuo* to yield the desired product (118 mg, 0.15 mmol, 100 %).

$^1$H NMR (400 MHz, 298 K, $CD_3CN$) **δ**: 3.77-3.73 (m, 2H), 3.69-3.63 (m, 4H), 3.63-3.54 (m, 10H), 3.39 (q, *J* = 6.3 Hz, 2H), 3.20-3.04 (m, 4H), 2.41 (t, *J* = 5.6 Hz, 2H); $^{19}$F NMR (377 MHz, 298 K, CD3CN) **δ**: -76.56 (TFA), -134.19 to -134.46 (m, 2F), -139.51 (ddt, *J* = 23.3, 9.2, 5.1 Hz, 2F), -139.90 (dt, *J* = 21.0, 10.5 Hz, 2F), -152.54 (td, *J* = 20.5, 3.8 Hz, 1F), -162.70 to -163.03 (m, 2F); HRMS m/z (EOS-TOF, +ve): calcd. for $[M+H]^+$ $C_{25}H_{28}F_9N_2O_5S$ 639.1575, found 639.1537.

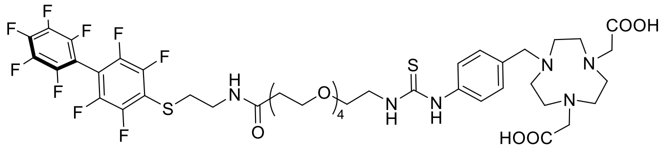


**PFBP-NODA (1)**

***PFBP-NODA (1)***: To a solution of PFBP-PEG$_4$-NH$_2$ (38 mg, 60 µmol) in anhydrous DMF (3 mL), under a nitrogen atmosphere, was added *N,N*-Diisopropylethylamine (34 µL, 0.19 mmol) and NCS-MP-NODA (20 mg, 50 µmol). The resulting solution was stirred at r.t. for 18 h. Volatiles were removed by passing a $N_2$ flow over the crude reaction before further drying *in vacuo* and the crude product purified *via* reversed-phase flash chromatography (C18, Biotage Sfar C18 Duo 100 Å 30 µm 12 g cartridge, A: 0.1 % TFA in $H_2O$ B: 0.1 % TFA in MeCN; 80 % A: 20 % B → 100 % B) and lyophilised to yield the desired product as an off-white oil (37.9 mg, 37 µmol, 73 %).

$^1$H NMR (400 MHz, 298 K, $CD_3CN$) **δ**: 7.57 (d, *J* = 8.1 Hz, 2H), 7.47 (d, *J* = 8.1 Hz, 2H), 7.05 (t, *J* = 6.0 Hz, 1H), 4.28 (s, 2H), 3.72 (s, 2H), 3.65-3.51 (m, 16H), 3.43 (s, 2H), 3.39-3.31 (m, 4H), 3.14 (t, *J* = 6.6 Hz, 4H), 3.09-2.94 (m, 4H), 2.74 (s, 4H), 2.70-2.59 (m, 2H), 2.34 (t, *J* = 6.0 Hz, 2H); $^{19}$F NMR (377 MHz, 298 K, $CD_3CN$) **δ**: -76.42 (TFA), -133.25 to -136.50 (m, 2F), -139.41 (dtq, *J* = 19.1, 8.9, 5.0 Hz, 2F), -139.81 to -140.00 (m, 2F), -152.55 (tt, *J* = 20.5, 3.3 Hz, 1F), - 162.43 to -163.11 (m, 2F); HRMS m/z (EOS-TOF, +ve): calcd. for $[M+H]^+$ $C_{43}H_{52}F_9N_6O_9S_2^+$ 1031.3094, found 1031.3138.

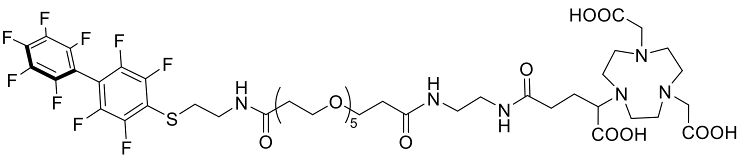

**PFBP-NODAGA (2)**

***PFBP-NODAGA (2)***: To a solution of Bis(NHS)PEG$_5$ (44 mg, 83 µmol) in anhydrous DMF (3 mL) under a nitrogen atmosphere, was slowly added a solution containing PFBP-NH$_2$ (**4.1**) (37 mg, 83 µmol) and *N,N*-Diisopropylethylamine (43 µL, 249 µmol) in anhydrous DMF (3 mL) over 30 min. The resulting solution was stirred at r.t. for 18 h after which, a solution containing NH$_2$-NODA-GA (45 mg, 108 µmol) and *N,N*-Diisopropylethylamine (57 µL, 332 µmol) in anhydrous DMF (2 mL) was added to the reaction. The resulting solution was stirred at r.t. for 18 h. Volatiles were then removed by passing a N$_2$ flow over the crude reaction before further drying *in vacuo* and the crude product purified *via* reversed-phase flash chromatography (C18, Biotage Sfar C18 Duo 100 Å 30 µm 12 g cartridge, A: 0.1 % TFA in H$_2$O B: 0.1 % TFA in MeCN; 80 % A: 20 % B → 100 % B) and lyophilised to yield the desired product as an off-white oil (29.8 mg, 27 µmol, 33 %).

$^1$H NMR (400 MHz, 298 K, CD$_3$CN) **δ**: 7.30 (br s, 1H), 7.03 (br s, 1H), 6.92 (br s, 1H), 3.89 (s, 2H), 3.69-3.62 (m, 6H), 3.59-3.51 (m, 16H), 3.38 (q, *J* = 6.5 Hz, 2H), 3.16 (t, *J* = 6.5 Hz, 6H), 3.03-2.92 (br m, 7 H), 2.84-2.78 (br m, 8H), 2.43 (t, *J* = 6.0 Hz, 2H), 2.35 (t, *J* = 6.0 Hz, 2H), 2.33-2.28 (m, 2H); $^{19}$F NMR (377 MHz, 298 K, CD3CN) **δ**: -76.44 (TFA), -134.27 (dd, *J* = 24.5, 11.3 Hz, 2F), -139.16 to -139.71 (m, 2F), -139.94 (dt, *J* = 21.9, 10.9 Hz, 2F), -152.57 (t, *J* = 20.4 Hz, 1F), -162.83 (t, *J* = 19.5 Hz, 2F); HRMS m/z (EOS-TOF, +ve): calcd. for $[M+H]^+$ $C_{45}H_{60}F_9N_6O_{14}S^+$ 1111.3745, found 1111.3789.

## Peptide Conjugation

For peptide conjugation reactions, the below precursor stock solutions were prepared:

***PFBP reagents***: **1**: 30 mM in DMSO, **2**: 50 mM in DMF; ***Peptides***: FCPF-TATE (**3**): 10 mM in H2O; ***Buffers***: 200 mM TCEP in 0.2 M phosphate pH 8.0, 0.2 M phosphate buffer at pH 8.0.

***General procedures for peptide conjugation reactions***: Peptide (1 mM in 0.2 M phosphate buffer) and PFBP-containing probe (5 mM in DMSO or DMF) were added to a solution containing TCEP (20 mM) and 0.2 M phosphate buffer (make up to 200 µL). The resulting solution / mixtures were then incubated at 30 °C or 37 °C with shaking on a thermomixer. Aliquots (40 µL) were taken after appropriate time points and quenched with 0.1 % TFA in 8:2 water: acetonitrile prior to LC-MS analysis.

***Synthesis of NODA-FCPF-TATE (4) and NODAGA-FCPF-TATE (5)***: Conjugation reaction was performed using the above protocol, but on a 5 mg scale (of **3**). Briefly, **3** (5 mg, 1 mM) was incubated with 5 eq. of the respective PFBP reagents (5 mM) and TCEP (20 mM) in 0.2 M phosphate buffer at 37 ˚C for 30 min. The reactions were then purified by prep-HPLC on an Agilent 1260 infinity II system (A: H$_2$O + 0.1 % TFA, B: MeCN + 0.1 % TFA, Gradient: 5-95 % B over 40 min). The fraction containing the desired product was lyophilised and redissolved in 0.2 M NH$_4$OAc (pH 5). 2,2-dipyridyldisulphide (5 eq.) was added to the solution and the resulting reactions incubated

at r.t. for 30 min. The reactions were then purified by prep-HPLC on an Agilent 1260 infinity II system (A: $H_2O$ + 0.1 % TFA, B: MeCN + 0.1 % TFA, Gradient: 5-95 % B over 40 min) to yield the desired products.

***NODA-FCPF-TATE (4)***: HRMS m/z (ESI-TOF, +ve): calcd for [M+2H]2+ 1277.9631, found 1277.9601.

***NODAGA-FCPF-TATE (5)***: HRMS m/z (ESI-TOF, +ve): calcd for [M+2H]2+ calc. 1317.9959, found 1318.0016.

## Radiochemistry

***[$^{18}$F]AlF-NODA-FCPF-TATE ([$^{18}$F]AlF-4):*** a solution containing **4** in DMSO (5 µL, 25 nmol), 2 mM $AlCl_3$ in 0.5 M sodium acetate at pH 4.2 (12.5 µL, 100 nmol), QMA purified [$^{18}$F]Fluoride (400-500 MBq, 100 µL) and DMSO (150 µL) was incubated in a sealed tube at 100 °C for 20 min. The reaction was then diluted in water (15 mL), trapped on a Sep-Pak C18 plus light cartridge (145 mg sorbent, preconditioned with 5 mL EtOH followed by 5 mL $H_2O$), washed with $H_2O$ (5 mL) and eluted with EtOH (0.5 mL). The product was analysed by C18 RP-HPLC: (0.1% TFA $H_2O$:MeCN 95:5 *v:v* to 5:95 *v:v* gradient, phenomenex, Gemini 5 µm C18 110 Å column, 1 mL/min flow rate): $t_R$ = 10.3 min.

***[$^{68}$Ga]Ga-NODAGA-FCPF-TATE ([$^{68}$Ga]Ga-5)***: [$^{68}$Ga]$Ga^{3+}$ was eluted from a GalliAd generator where $^{68}$Ge was attached to a tin dioxide column using 0.1 M HCl. ~430 MBq [$^{68}$Ga]$Ga^{3+}$ is typically obtained in 1.1 mL and is used without further purification. A solution containing **5** in DMSO (150 µL, 57 nmol), [$^{68}$Ga]$Ga^{3+}$ (125 µL, 56 MBq) and 2 M $NH_4OAc$ (62.5 µL, pH 6) were incubated at 37 °C for 15 min. The reaction was diluted with water (5 mL), trapped on a Sep-Pak C18 Plus light cartridge (145 mg sorbent, preconditioned with 5 mL EtOH followed by 5 mL $H_2O$), washed with $H_2O$ (5 mL) and eluted with EtOH (1 mL). The product was analysed by C18 RP-HPLC: (0.1% TFA $H_2O$:MeCN 95:5 *v:v* to 5:95 *v:v* gradient, Agilent ZORBAX Eclipse XDB-C18 column, 1 mL/min flow rate): $t_R$ = 13.7 min. Radiochemical purity (RCP): >99 %; decay corrected (d.c.) radiochemical yield (RCY): 49.7 ± 8.5 % (n=3)
***For in vitro and in vivo analysis:*** A solution containing **5** in DMSO (10 µL, 3.8 nmol), [$^{68}$Ga]$Ga^{3+}$ (70 µL, 30 MBq) and 2 M $NH_4OAc$ (5 µL, pH 6) were incubated at 90 °C for 10 min. The reaction was diluted with water (5 mL), trapped on a Sep-Pak C18 Plus light cartridge (145 mg sorbent, preconditioned with 5 mL EtOH followed by 5 mL $H_2O$), washed with $H_2O$ (5 mL) and eluted with EtOH (1 mL). Bulk of the EtOH was removed over a stream of $N_2$ gas and the product reconstituted in PBS for subsequent biological evaluations. RCP: >99 %; d.c. RCY: 49.5 ± 3.2 % (n=4). Apparent molar activity: 3.05 GBq $\mu mol^{-1}$.

***$LogD_{7.4}$ measurements***: **[$^{18}$F]AlF-4** or **[$^{68}$Ga]Ga-5** (3-5 MBq) were added to a mixture of PBS (0.5 mL) and n-octanol (0.5 mL) and shaken at r.t. for 10 min after which the mixtures were centrifuged (13,000 g 5 min) to separate the two layers. Aliquots (6 x 100 µL each) from the PBS and n-octanol layers were removed and counted for radioactivity in a gamma counter. **[$^{18}$F]AlF-4**: $LogD_{7.4}$ = 0.40 ± 0.10; **[$^{68}$Ga]Ga-5**: $LogD_{7.4}$ = -0.40 ± 0.03.

## *In vitro* Analysis

***Stability of [$^{18}$F]AlF-4***: **[$^{18}$F]AlF-4** (3-5 MBq) was incubated at 37 °C in RPMI media (500 µL). Aliquots of the solutions were sampled at 1, 2, 3, 4 and 5 h and diluted in $H_2O$ for C18 RP-HPLC analysis. The diluted solutions were centrifuged at 13,000 g for 5 min prior to analysis.

***Stability of [$^{68}$Ga]Ga-5***: A sample of **[$^{68}$Ga]Ga-5** was added to serum (with or without 5 mM reduced glutathione), in a ratio of one part radiolabelled solution to four parts of serum by volume and incubated at 37 °C. Aliquots were taken for SE-HPLC analysis at t = 1 h, 2 h, 3 h, 4 h and 5 h.

***Cell culture***: The rat cancer cell line AR42J (cultured in RPMI-1640) was purchased from American Type Culture Collection. Growth media were supplemented with 10 % foetal bovine serum (FBS), 100 units/mL penicillin, 100 µg/mL streptomycin, and 2 mM ʟ-glutamine. Cell lines were harvested twice weekly using a formulation of 0.25 % trypsin/0.53 mM EDTA in Phosphate Buffered Salt (PBS) Solution without calcium and magnesium, which was then neutralised with the appropriate medium containing FBS. Cells were maintained in a humidified chamber containing 5 % $CO_2$ at 37 °C.

***SSTR2 in vitro binding studies***: **[$^{68}$Ga]Ga-5** was prepared as per above. The radioconjugate was then diluted with HBSS to a final concentration of 68 nM prior to uptake studies. Once harvested after trypsinisation, AR42J cells were washed once with PBS and resuspended in RPMI with 10 % FBS to give 1 x $10^6$ cells per well in 500 µL, in 6-well cell culture dishes. Cells were incubated overnight and resuspended in fresh RPMI with 10 % FBS prior to radiotracer addition. **[$^{68}$Ga]Ga-5** (100 µL, 68 nM in PBS) were added to each well. For blocking conditions, unmodified octreotide (14 µL of 380 µM in saline) was added to the wells and incubated at r.t. for 5 min prior to addition of the radioconjugate. The well plates were incubated at 37 °C for 5, 15, 30 or 60 min in triplicate. Unbound free radiotracer was collected, with the supernatant and PBS washes (500 µL x 2) combined for this fraction. Cell-surface bound tracer fraction was collected through washing with glycine buffer (pH 2.8, 500 µL x 1). Finally, the internalised fraction was collected through washing with 1 M NaOH (500 µL x 1) for 5 min, followed by PBS wash (500 µL x 1). The radioactivity associated with each fraction were then counted using a gamma counter and presented as percentage of added activity. Data were presented as mean ± SD. Comparisons were made using paired two-tailed t-tests using the GraphPad Prism 9.0 software.

## *In vivo* Analysis

All animal experiments were ethically reviewed by an Animal Welfare & Ethical Review Board at King's College London and carried out in accordance with the Animals (Scientific Procedures) Act 1986 (ASPA) UK Home Office regulations governing animal experimentation.

***PET/CT imaging and reconstruction:*** 9-month-old female BALB/c mice (22-27 g) n=3/group were obtained from Charles Rivers Laboratories. Animals were housed in ventilated cages, were given food and water ad libitum. Preclinical PET/CT images were acquired using a NanoScan PET/CT scanner (Mediso, Budapest, Hungary) with

mice under 0.8-1.5% isoflurane in oxygen anaesthesia and warmed to 37 °C for the duration of the experiment. Mice were administered **[$^{68}$Ga]Ga-5** (~2-3 µg, ~0.8-1.5 MBq) in 200 µL of PBS *via* intravenous tail injection. Dynamic PET scans were acquired for up to 1 h post injection, followed by a CT scan for anatomical visualisation (480 projections; helical acquisition; 55 kVp; 600 ms exposure time). PET/CT datasets were reconstructed using a Monte Carlo-based full-3D iterative algorithm (TeraTomo, Mediso) with 4 iterations, 6 subsets, and 0.4 mm isotropic voxel size. Images were co-registered and analysed using VivoQuant v.3.0 (Invicro). Regions of interest (ROIs) were delineated for PET activity quantification in specific organs. Uptake in each ROI was expressed as a percentage of injected dose per gram of tissue (% ID/g).

***Biodistribution studies:*** Following PET/CT imaging study, mice (n=4/group, one mouse from each group was not subjected to PET/CT imaging) were culled by cervical dislocation, organs were dissected, weighed, and gamma-counted along with standards prepared from the corresponding sample of injected radiolabelled conjugates. Conjugate uptake was calculated as a percentage of injected dose per gram (% ID/g) of tissue.

# Supplementary Figures

## 1.1 PFBP-NODA (1) and PFBP-NODAGA (2) Characterisation Data

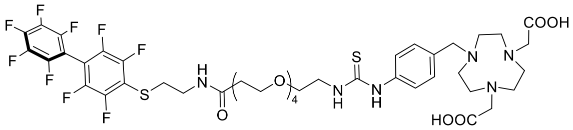


**PFBP-NODA**

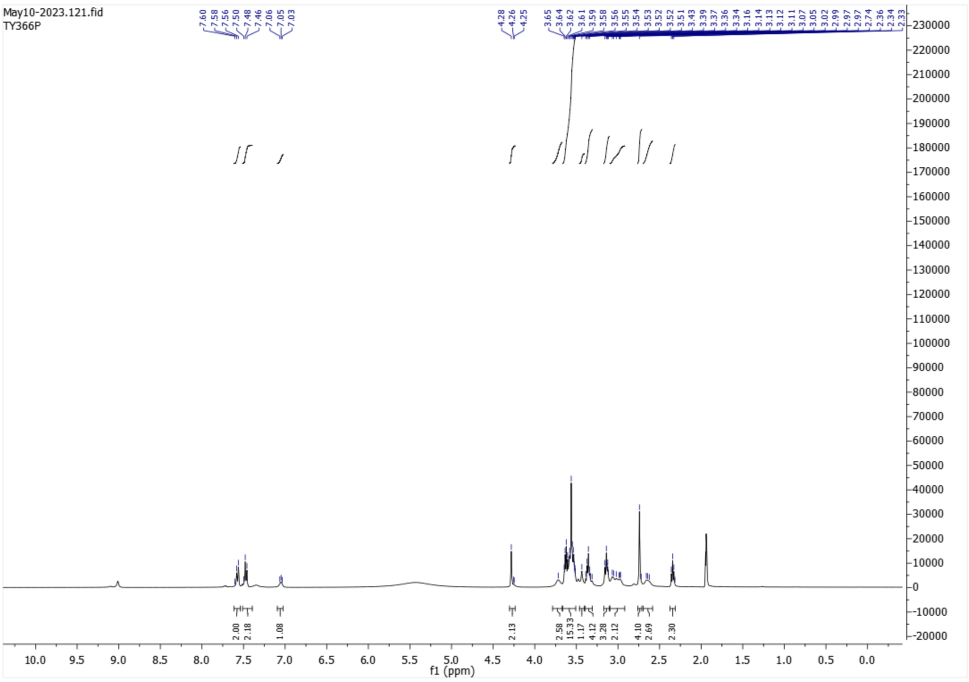


Figure S1 $^{1}H$ NMR spectrum of **1** (400 MHz, 298 K, $CD_3CN$).

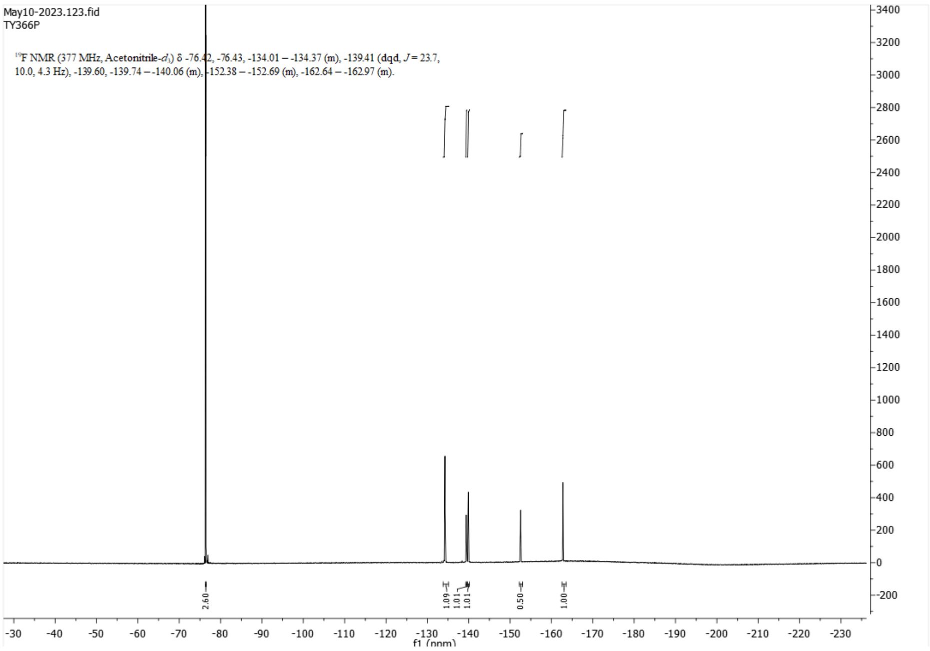


Figure S2 $^{19}F\{^{1}H\}$ NMR spectrum of **1** (377 MHz, 298 K, $CD_3CN$).

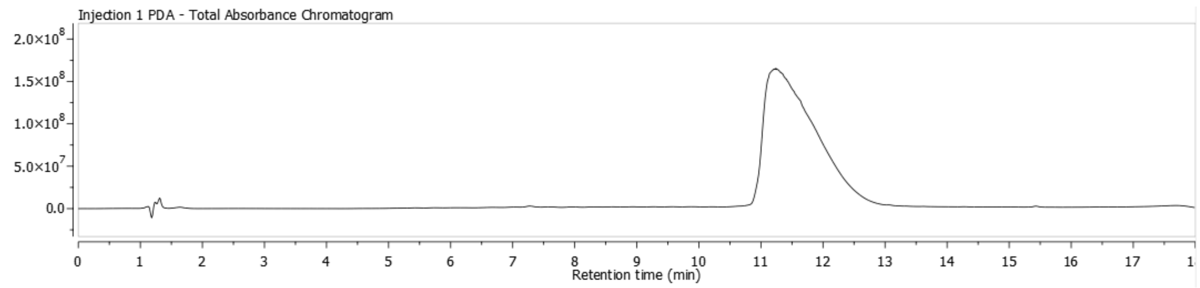


Figure S3 Total absorbance chromatogram from LC-MS analysis of **1**.

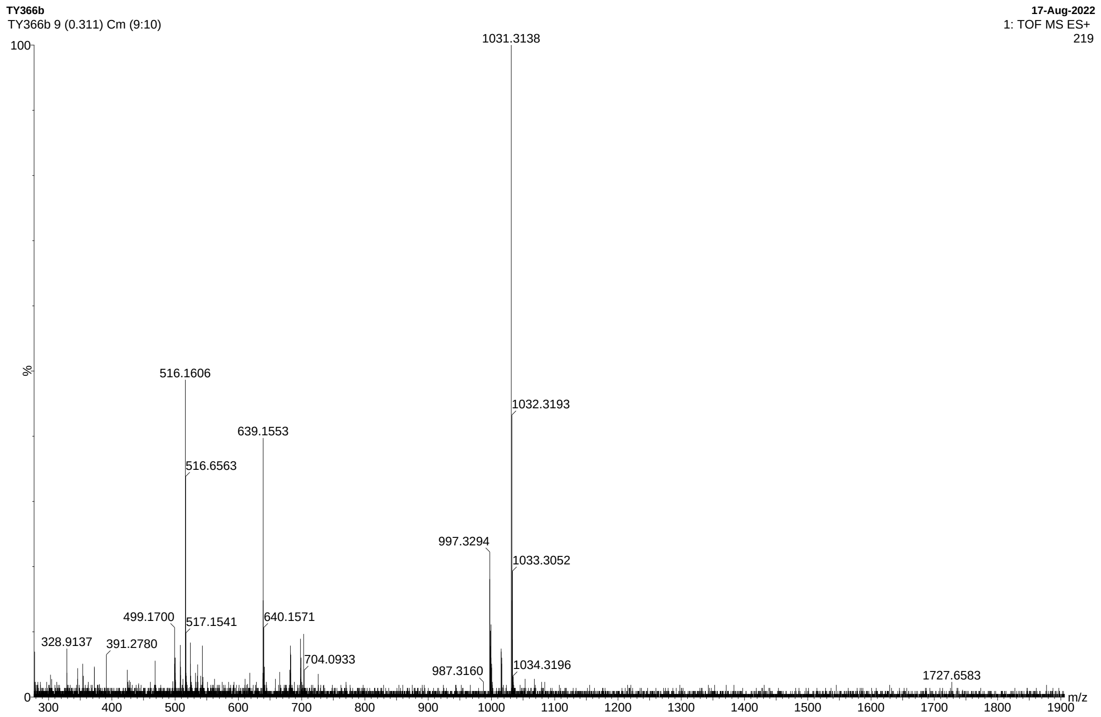


Figure S4 High resolution mass spectrum of **1** (EOS-TOF, +ve): calcd. for $[M+H]^+$ $C_{43}H_{52}F_9N_6O_9S_2^+$ 1031.3094, found 1031.3138

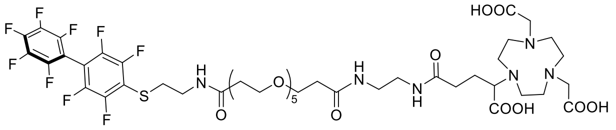


**PFBP-NODAGA**

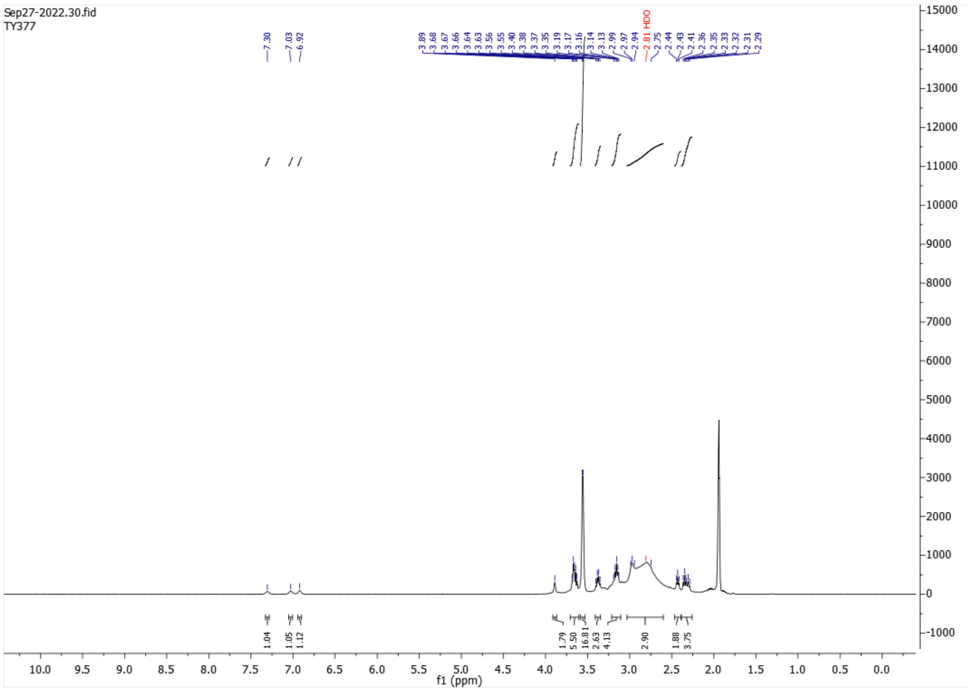


Figure S5 $^{1}H$ NMR spectrum of **2** (400 MHz, 298 K, $CD_3CN$).

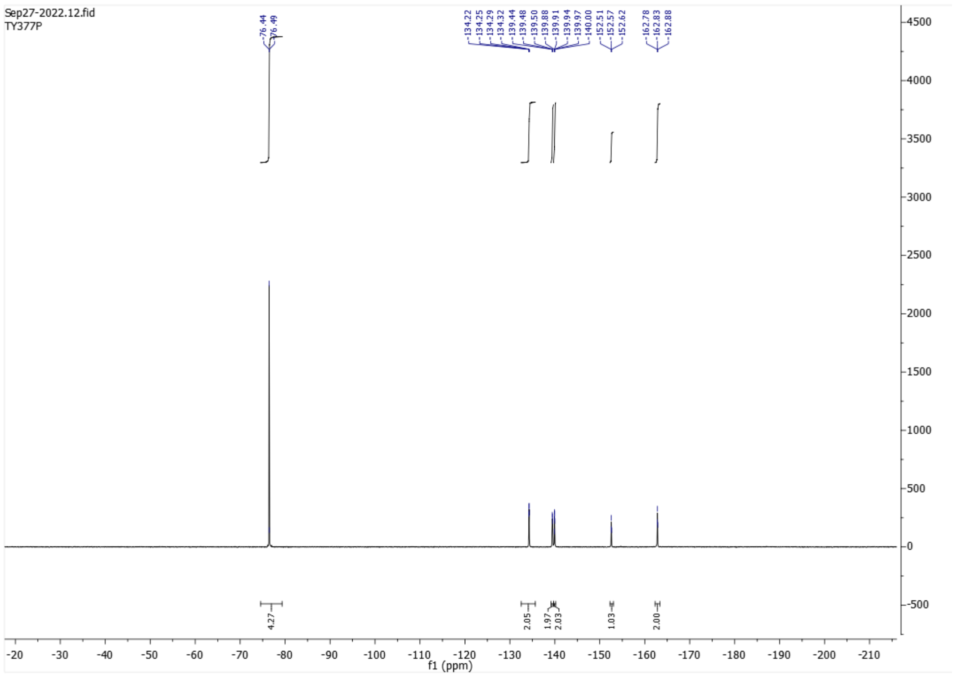


Figure S6 $^{19}F\{^{1}H\}$ NMR spectrum of **2** (377 MHz, 298 K, $CD_3CN$).

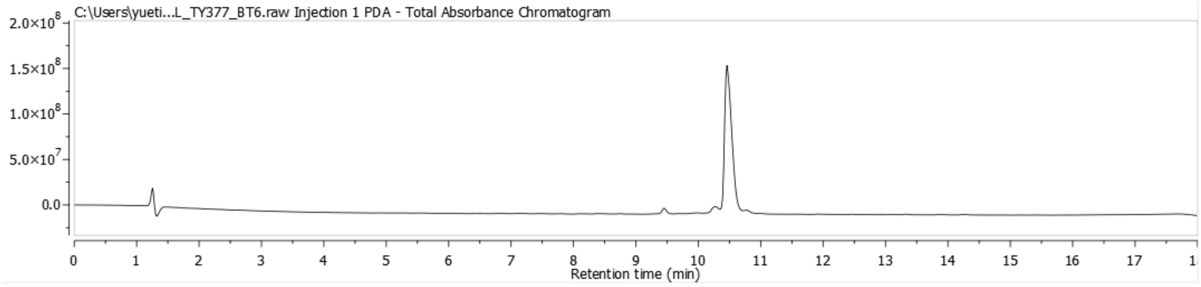


Figure S7 Total absorbance chromatogram from LC-MS analysis of **2**.

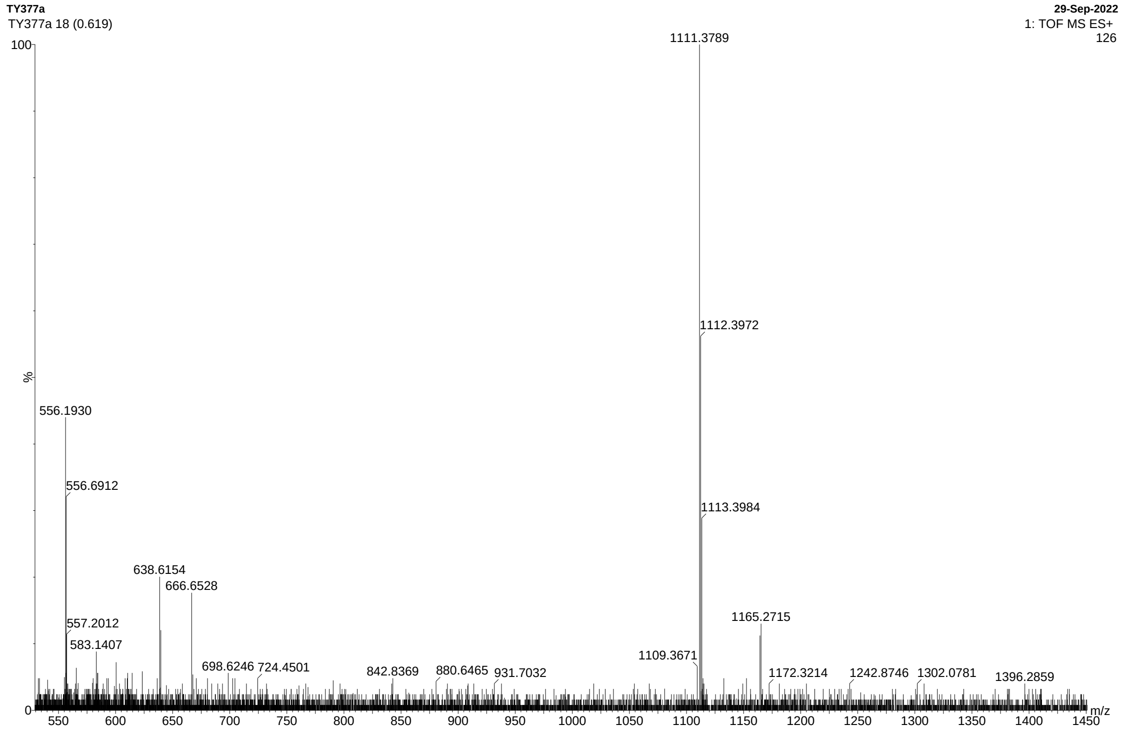


Figure S8 High resolution mass spectrum of **2** (EOS-TOF, +ve): calcd. for $[M+H]^+$ $C_{45}H_{60}F_9N_6O_{14}S^+$ 1111.3745, found 1111.3789.

## 1.2 Peptide Conjugation

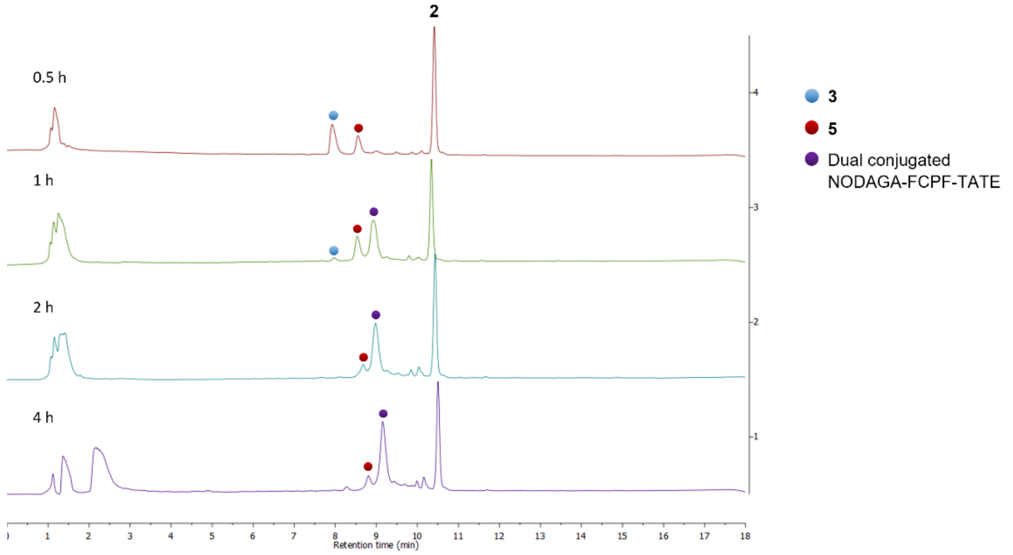


Figure S9: Conjugation reaction between **2** and **3**: RP-HPLC-MS total absorbance chromatogram of the reaction over 4 h

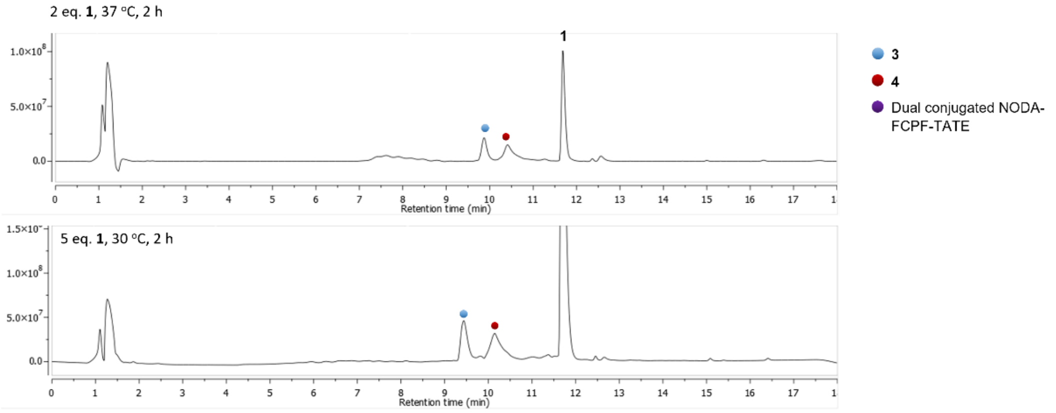


Figure S10: Conjugation reaction between **1** and **3**: RP-HPLC-MS total absorbance chromatograms of the reactions at 2 h employing 2 eq. of **1** at 37 °C (top) and 5 eq. of **1** at 30 °C (bottom). Formation of dual-conjugated species could be effectively minimised using these conditions.

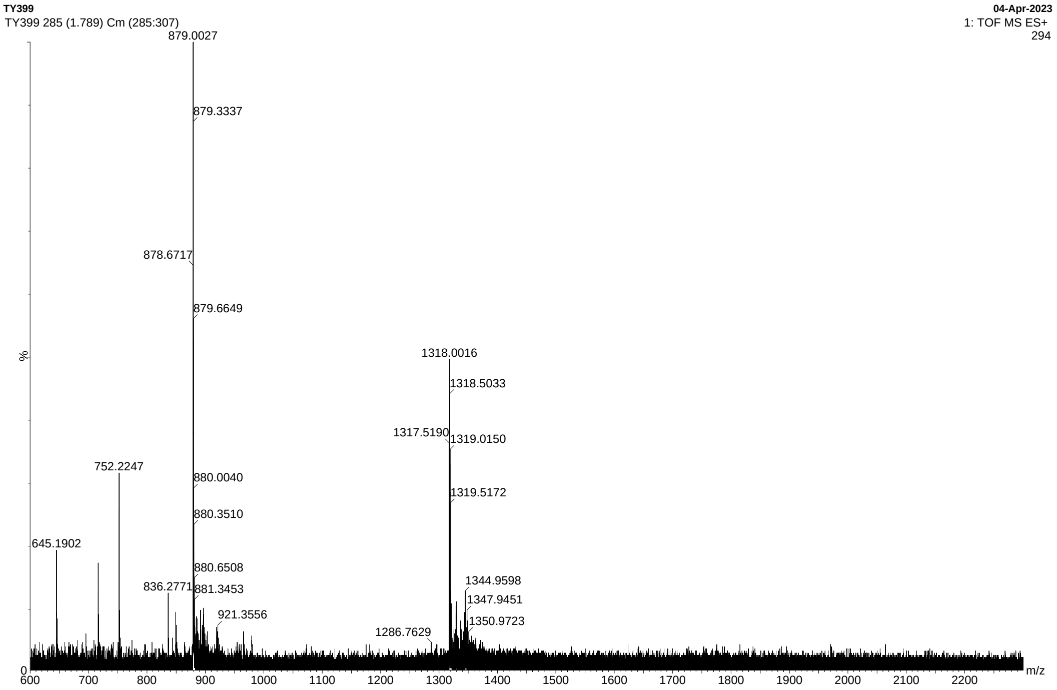


Figure S11: High resolution mass spectrum of **5** (EOS-TOF, +ve): calcd for [M+2H]2+ calc. 1317.9959, found 1318.0016.

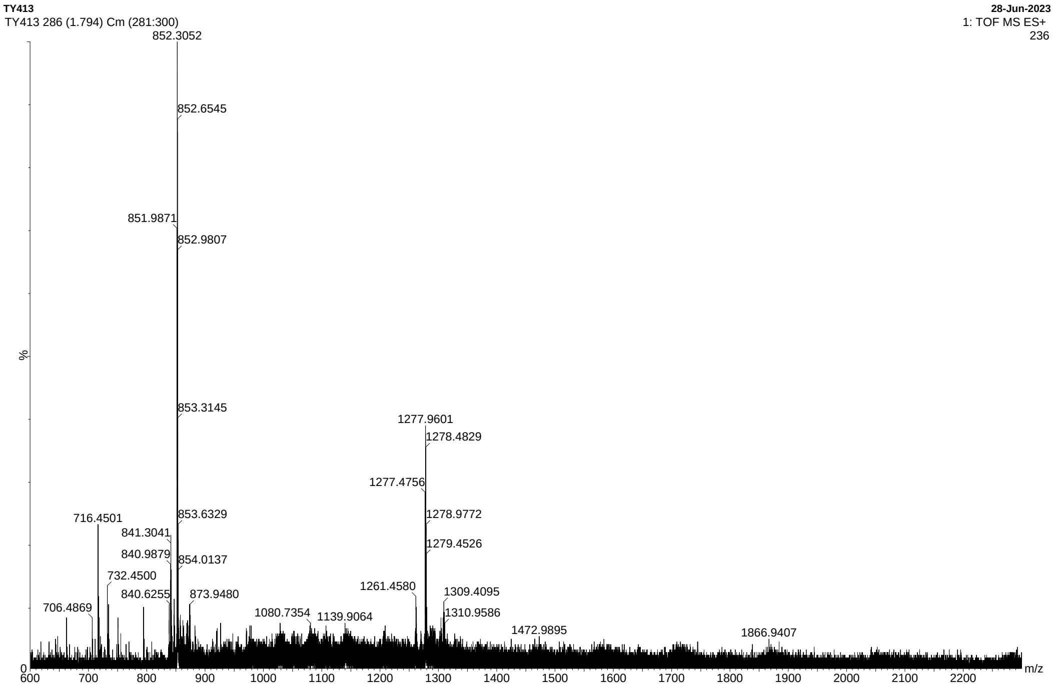


Figure S12: High resolution mass spectrum of **4** (EOS-TOF, +ve): calcd for [M+2H]2+ 1277.9631, found 1277.9601.

## BioConfirm Protein Digest Report

Agilent Trusted Answers

**Sequence Details**

*FCPF-Octreotate Sequence*

**Coverage Map**

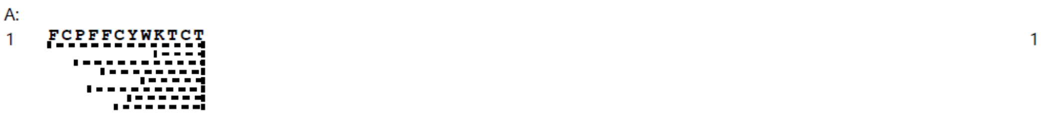


**Biomolecule Summary**

| Biomol | Seq Loc | Mods | Var Mods | Score (Bio) | RT | Height | Mass | Tgt Mass | Diff (ppm) |
|---|---|---|---|---|---|---|---|---|---|
| 18 | A(8-12) | | | | 5.523 | 130934 | 637.2885 | 637.2894 | -1.37 |
| 9 | A(9-12) | | | | 5.523 | 203079 | 451.2097 | 451.2101 | -0.80 |
| 25 | A(4-12) | | | | 5.523 | 106650 | 1197.4963 | 1197.4987 | -1.99 |
| 28 | A(6-12) | | | | 5.523 | 97567 | 903.3614 | 903.3619 | -0.50 |
| 1 | A(1-12) | | 1*PFBP-PEG-NODA | | 5.523 | 4240826 | 2554.9204 | 2554.9244 | -1.55 |
| 27 | A(7-12) | | | | 5.523 | 98607 | 800.3531 | 800.3527 | 0.44 |
| 16 | A(5-12) | | | | 5.524 | 132751 | 1050.4288 | 1050.4303 | -1.48 |
| 10 | A(3-12) | | | | 5.524 | 186146 | 1294.5499 | 1294.5515 | -1.19 |

Figure S13 HPLC-MS/MS peptide mapping analysis of **4** indicating a single modification at Cys2.

## BioConfirm Protein Digest Report

Agilent Trusted Answers

**Sequence Details**

*FCPF-Octreotate Sequence*

**Coverage Map**

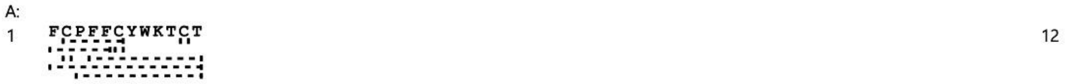


**Biomolecule Summary**

| Biomol | Seq Loc | Mods | Var Mods | Score (Bio) | RT | Height | Mass | Tgt Mass | Diff (ppm) |
|---|---|---|---|---|---|---|---|---|---|
| 1612 | A(2-6) | | 2*PFBP-PEG-NODA(+1010.295295)A2A6 | 2.88 | 7.092 | 65524 | 2635.8348 | 2635.8091 | 9.73 |
| 1927 | A(1-5) | | 1*PFBP-PEG-NODA(+1010.295295)A2 | 12.25 | 7.433 | 2433 | 1669.5838 | 1669.5731 | 6.43 |
| 2330 | A(2-2)/ A(6-6)/ A(11-11) | | 1*PFBP-PEG-NODA(+1010.295295)A2, 1*PFBP-PEG-NODA(+1010.295295)A6, 1*PFBP-PEG-NODA(+1010.295295)A11 | 29.71 | 8.048 | 3347 | 1131.3142 | 1131.3150 | -0.72 |
| 2574 | A(1-12) | | 2*PFBP-PEG-NODA(+1010.295295)A2A6 | 29.10 | 8.424 | 108076 | 3565.2235 | 3565.2197 | 1.07 |
| 2664 | A(4-12) | | 1*PFBP-PEG-NODA(+1010.295295)A6 | 21.96 | 8.495 | 2237 | 2207.7860 | 2207.7940 | -3.63 |
| 2673 | A(3-12) | | 1*PFBP-PEG-NODA(+1010.295295)A6 | 29.93 | 8.496 | 4858 | 2304.8475 | 2304.8468 | 0.31 |
| 2769 | A(1-12) | | 2*PFBP-PEG-NODA(+1010.295295)A2A6 | 29.98 | 8.504 | 296819 | 3565.2191 | 3565.2197 | -0.17 |
| 2917 | A(1-12) | | 2*PFBP-PEG-NODA(+1010.295295)A2A6 | 29.91 | 8.743 | 4717 | 3565.2185 | 3565.2197 | -0.33 |

Figure S14 HPLC-MS/MS peptide mapping analysis of dual-modified NODA-FCPF-TATE indicating modifications at Cys2 and Cys6.

## 1.3 Radiochemistry

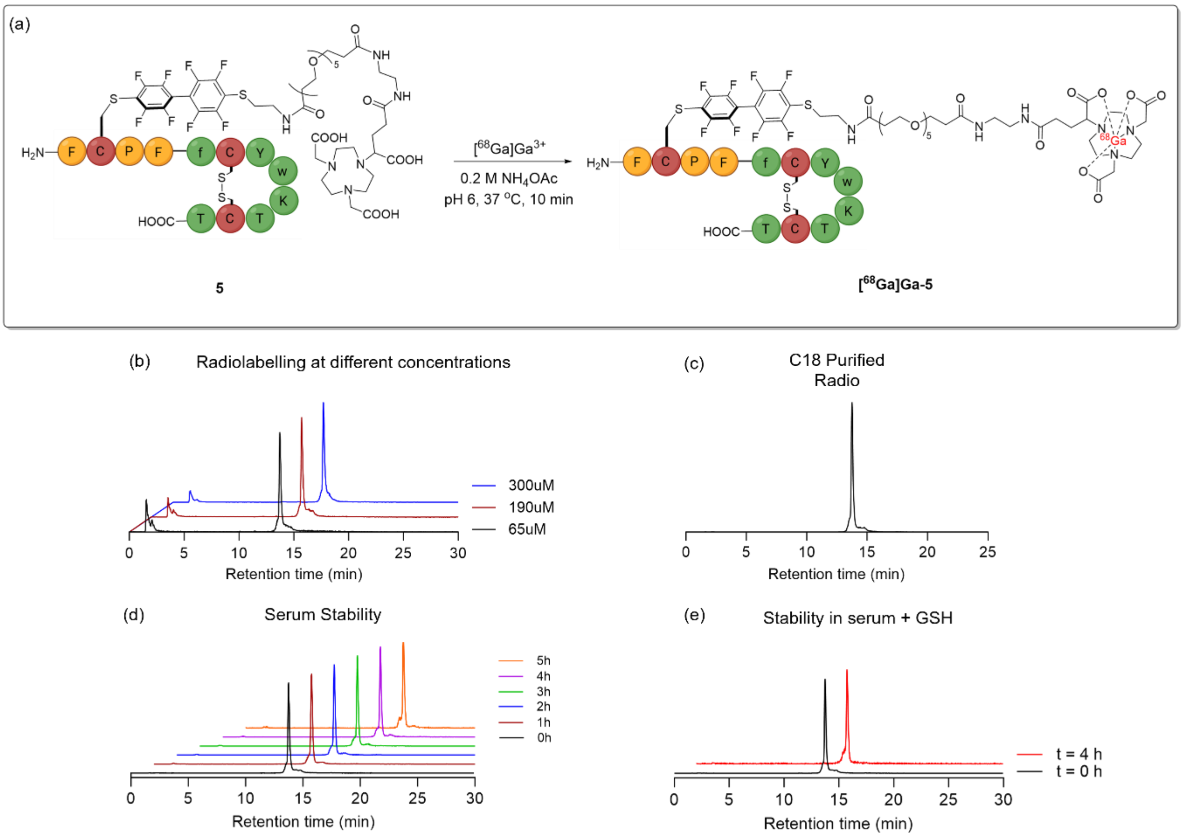


Figure S15 (a) [$^{68}$Ga]Ga$^{3+}$ radiolabelling of **5**. C18 RP-HPLC radiochromatogram of (b) radiolabelling efficiencies at 65, 190 and 300 µM, (c) purified **[$^{68}$Ga]Ga-5**, (d) human serum stability of the radiotracer up to 5 h, (e) stability of the radiotracer in human serum and 5 mM reduced glutathione (GSH).

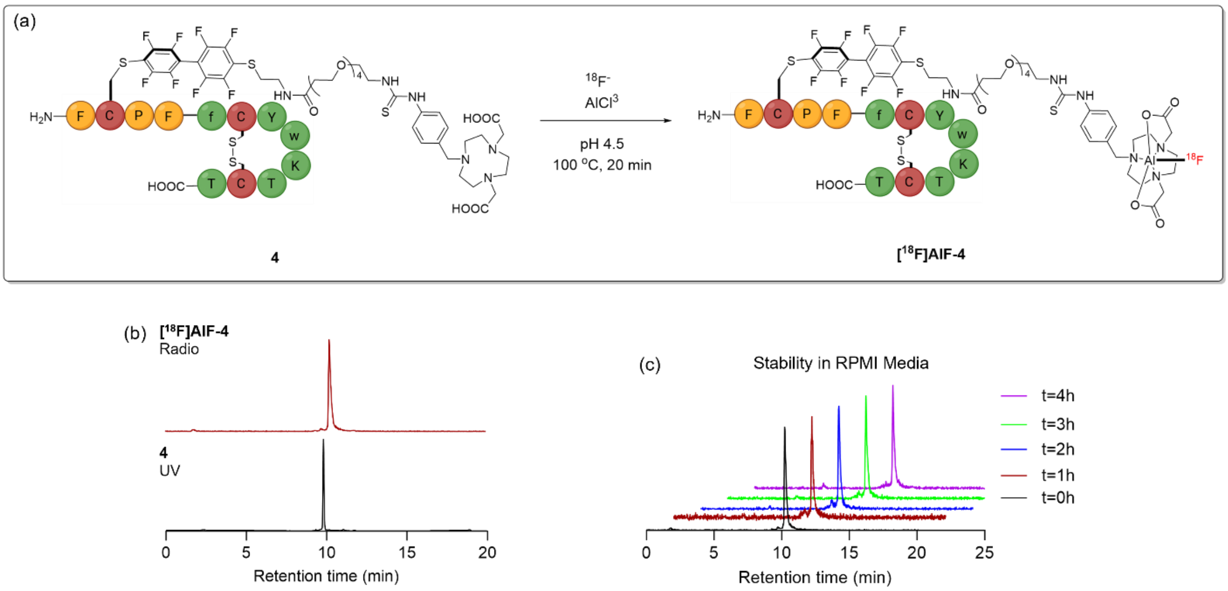


Figure S16 (a) [$^{18}$F]AlF-radiolabelling of **4**. (b) C18 RP-HPLC radiochromatogram of the purified tracer and UV chromatogram of the precursor. (c) C18 RP-HPLC analysis of the radiotracer in RPMI Media over 4 h.

## 1.4 *In vitro* cell uptake

Table S1 *In vitro* cell binding study of **[$^{68}$Ga]Ga-5** with SSTR2-positive AR42J cells and under blocking conditions through addition of 200-fold unmodified octreotide. Supplementary to main text Figure 3.

| Time (min) | Internalised **[$^{68}$Ga]Ga-5** (% added activity) | | Internalised **[$^{68}$Ga]Ga-5** + Blocked (% added activity) | | Total uptake **[$^{68}$Ga]Ga-5** (% added activity) | | Total uptake **[$^{68}$Ga]Ga-5**+ Blocked (% added activity) | |
|---|---|---|---|---|---|---|---|---|
| | Mean (n=3) | S.D. | Mean (n=3) | S.D. | Mean (n=3) | S.D. | Mean (n=3) | S.D. |
| 5 | 4.700 | 0.900 | 1.533 | 0.493 | 6.867 | 0.971 | 3.933 | 0.493 |
| 15 | 9.133 | 0.643 | 2.567 | 0.907 | 11.533 | 0.404 | 4.433 | 0.777 |
| 30 | 12.933 | 0.115 | 4.367 | 3.179 | 17.467 | 3.406 | 6.033 | 2.950 |
| 60 | 22.667 | 3.302 | 3.333 | 0.404 | 25.267 | 3.002 | 5.400 | 0.458 |

## 1.5 *Ex vivo* biodistribution

Table S2 *Ex vivo* biodistribution data of **[$^{68}$Ga]Ga-5** in BALB/c healthy mice at 1 h p.i. (n=4). Supplementary to main text Figure 4.

| | **[$^{68}$Ga]Ga-5** (%ID $g^{-1}$) | | **[$^{68}$Ga]Ga-5** + Blocked (%ID $g^{-1}$) | |
|---|---|---|---|---|
| | Mean (n=4) | S.D. | Mean (n=4) | S.D. |
| Blood | 27.243 | 3.520 | 16.708 | 6.713 |
| Heart | 7.895 | 0.933 | 5.848 | 2.052 |
| Lungs | 10.610 | 2.709 | 14.580 | 2.472 |
| Liver | 54.588 | 22.473 | 32.820 | 1.565 |
| Spleen | 20.768 | 11.763 | 11.430 | 3.374 |
| Pancreas | 3.493 | 1.064 | 2.645 | 0.732 |
| Stomach | 2.168 | 1.183 | 3.313 | 0.998 |
| Kidneys | 9.575 | 5.813 | 8.645 | 2.248 |
| Adrenal glands | 6.453 | 2.281 | 5.300 | 1.470 |
| Upper GI | 5.640 | 2.142 | 6.950 | 6.430 |
| Lower GI | 2.760 | 1.742 | 2.053 | 0.323 |
| Muscle | 0.690 | 0.334 | 1.260 | 0.254 |
| Bone | 0.950 | 0.458 | 1.640 | 0.476 |
| Pituitary gland | 6.040 | 4.823 | 3.103 | 2.698 |
| Brain | 1.290 | 1.215 | 0.418 | 0.135 |

## 1.6 Dynamic PET/CT Imaging time-activity curves

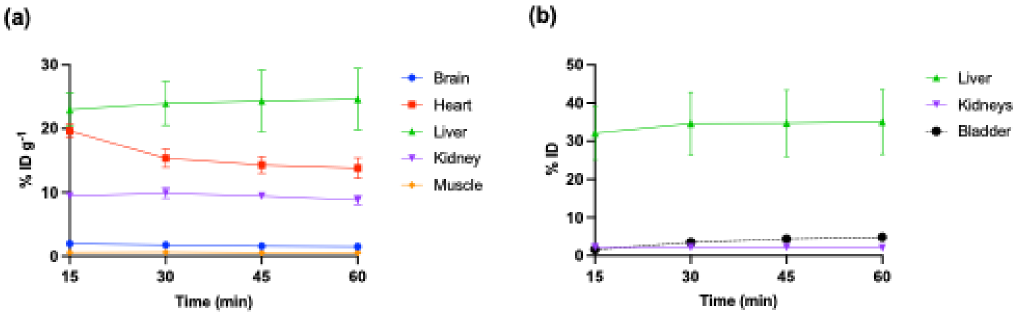


Figure S17 Regions of interest were selected on VivoQuant (inviCRO, LLC, Boston, MA), and percentages of (a) injected dose per milliliter (% ID/mL) and (b) injected dose (% ID) were calculated for selected organs/tissues.
